# Association of *LPP* and *ZMIZ1* Gene Polymorphism with Celiac Disease in Subjects from Punjab, Pakistan

**DOI:** 10.3390/genes15070852

**Published:** 2024-06-27

**Authors:** Sumaira Zulfiqar, Amna Fiaz, Waqas Ahmed Khan, Misbah Hussain, Ansar Ali, Nadeem Ahmed, Basharat Ali, Muhammad Adnan Masood

**Affiliations:** 1 Department of Biotechnology, Faculty of Sciences, University of Sargodha, Sargodha 40162, Pakistanmisbah.hussain@uos.edu.pk (M.H.);; 2 Centre of Excellence in Molecular Biology, University of the Punjab, Lahore 42000, Pakistan; 3 Department of Family Medicine, University of Health Sciences, Lahore 42000, Pakistan; 4 Department of Medicine, Niazi Medical & Dental College Sargodha, Sargodha 40100, Pakistan

**Keywords:** celiac disease, gastrointestinal, *LPP*, rs1464510, rs1250552, *ZMIZ1*

## Abstract

Celiac disease (CD) is a complicated autoimmune disease that is caused by gluten sensitivity. It was commonly believed that CD only affected white Europeans, but recent findings show that it is also prevailing in some other racial groups, like South Asians, Caucasians, Africans, and Arabs. Genetics plays a profound role in increasing the risk of developing CD. Genetic Variations in non-*HLA* genes such as *LPP*, *ZMIZ1*, *CCR3*, and many more influence the risk of CD in various populations. This study aimed to explore the association between LPP rs1464510 and ZMIZ1 rs1250552 and CD in the Punjabi Pakistani population. For this, a total of 70 human subjects were selected and divided into healthy controls and patients. Genotyping was performed using an in-house-developed tetra-amplification refractory mutation system polymerase chain reaction. Statistical analysis revealed a significant association between LPP rs1464510 (χ^2^ = 4.421, *p* = 0.035) and *ZMIZ1* rs1250552 (χ2 = 3.867, *p* = 0.049) and CD. Multinomial regression analysis showed that LPP rs1464510 A allele reduces the risk of CD by ~52% (OR 0.48, CI: 0.24–0.96, 0.037), while C allele-carrying subjects are at ~2.6 fold increased risk of CD (OR 3.65, CI: 1.25–10.63, 0.017). Similarly, the *ZMIZ1* rs1250552 AG genotype significantly reduces the risk of CD by 73% (OR 0.26, CI: 0.077–0.867, *p* = 0.028). In summary, Genetic Variations in the LPP and *ZMIZ1* genes influence the risk of CD in Punjabi Pakistani subjects. LPP rs1464510 A allele and *ZMIZ1* AG genotype play a protective role and reduce the risk of CD.

## 1. Introduction

Celiac disease (CD) is a complex, autoimmune inflammatory disease affecting 3 million humans globally [1]. Subjects suffering from CD are very sensitive to gluten protein, mainly present in wheat, rye, and barely. Upon exposure to gluten, intestinal cells are damaged due to the overactivation of the immune system, which results in diarrhea, fatigue, weight loss, and many other undesirable symptoms. The incidence rate of CD varies among countries; European countries have a higher CD prevalence as compared to the Asia–Pacific region. These differences in CD prevalence could be due to ethnic differences in genetic factors and per-capita wheat consumption [2,3]. CD was often believed to only affect white Europeans, but new findings show that it is also prevailing in other racial groups, including South Asians, Caucasians, Africans, and Arabs [4]. 

Almost 0.7% of people in the United States and 1% of people in Europe are affected by CD [5,6]. Rapidly changing dietary choices and lifestyles, along with advancements in diagnostic techniques, could be the reasons for the increasing incidence of CD. According to recent studies, the frequency of CD in Middle Eastern Arab countries ranges from 0.6 percent to 1.1 percent [7]. 

Patients with CD exhibit a broad range of symptoms, including intestinal and non-intestinal symptoms [8]. However, intestinal symptoms are more commonly reported [9]. CD is also one of the causes of malabsorption due to damaged intestinal cell lining, which can result in reduced growth and development in children; however, these side effects are less noticeable in adults.

Intake of a gluten-free diet is the only cure/therapy for CD [10]. Although a gluten-free diet effectively manages CD and helps improve the intestinal cell lining, some patients do not respond well to this dietary intervention and develop CD-related complications like intestinal adenocarcinoma, T-cell lymphoma, and refractory sprue [11]. 

Specific serological tests, histological analysis of duodenal biopsies, and a gluten-free diet are all necessary for the diagnosis of CD [7]. Genetic testing for HLA susceptibility is increasingly used to diagnose celiac disease and assess family member risk [12]. Observance of a strict gluten-free diet for life is the only known cure. Most people who follow a gluten-free diet observe that their symptoms return to normal after four weeks of dietary intervention [13]. Serologic normalization of tissue transglutaminase antibodies, which can take a month to more than a year, is generally preceded by symptom improvement (resolution of diarrhea or reduction of abdominal discomfort), which is subsequently followed by improvement in histologic findings. As was already said, not everyone’s intestines heal [14].

The innate immune system and adaptive immune systems are both involved in CD pathogenesis. Protein peptides from gluten enter the intestinal lamina propria [15], where they have decayed, been identified by antigen-presenting cells (APCs) through *HLA* class II molecules, and ultimately result in an abnormal CD4+ T cell-mediated immunological response [9]. An integral part of CD pathophysiology is the formation of the peptide-*HLA* complex on APCs, which controls the transcription, configuration, and signaling preferences for the events implicated in celiac disease [16].

Along with environmental/dietary factors, genetic factors also contribute to the onset of CD [17]. Approximately ~40% of CD risk is associated with the human leukocyte antigen (*HLA)* class II haplotype DQ2 or DQ8 [18]. It is well reported that intestinal microbiome composition changes due to HLA haplotypes, and this dysbiosis stimulates gluten sensitivity [17]. Till now, 39 non-*HLA* genes have been identified to increase the risk of CD [9]. Some of the non-*HLA* genes overlap with other pathologies like Crohn’s disease, type 1 diabetes, rheumatoid arthritis, and juvenile idiopathic arthritis. Lipoma’s preferred partner protein is encoded by one of the non-*HLA* genes, *LPP*. Lipoma preferred partner (*LPP*) protein is localized in the cell periphery and interacts with paxillin in focal adhesions [19]. It is also reported that *LPP*-associated paxillin is more frequently observed in focal adhesions of enterocytes in CD patients as compared to controls [20]. Thus, alterations in cell shape and arrangement of the cytoskeleton in celiac enterocytes could be triggered by *LPP*, which is encoded by the *LPP* gene [20]. Another protein that is a member of the protein inhibitor of the activated STAT (PIAS) family of proteins is encoded by the *ZMIZ1* gene, which is considered a risk gene for vitiligo in the Chinese population [21] owing to its role in the proliferation and migration of melanocytes [22]. Zinc finger MIZ-type containing 1 (*ZMIZ1*) encoded by the *ZMIZ1* gene regulates the activity of various transcription activators for smad3/4, Notch1, p53, and androgen receptors. 

It is also reported that genetic variations in *LPP* and *ZMIZ1* can influence their function and increase the risk of CD and other diseases [23,24]. In recent years, due to advancements in technology, several loci have been studied for their association with CD; among these, *LPP* and *ZMIZ1* have been in focus [25,26]. However, the genetic-based association of *LPP* and *ZMIZ1* genes with increased CD risk is inconsistent [27,28,29]. The prevalence of CD in Pakistan is unknown; however, according to clinicians, CD is quite common among children and adults in our clinical setting [30].

Apart from this, CD is the least studied pathology in Pakistan [31]. Data obtained from several databases showed that only a few articles from Pakistan have been published, and most of these are published in national journals. These articles primarily focus on the clinical side of CD and the challenges of gluten-free diets. No article has been published on the genetics of Pakistani CD patients. Thus, the current study aimed to check the association of *LPP* and *ZMIZ1* genetic variations with the risk of CD in Pakistani patients. 

## 2. Materials and Methods

### 2.1. Sample Collection and DNA Extraction

This study was approved by the institutional (University of Sargodha, Pakistan) ethics review committee. The approval code is SU/ORIC/801 (data: 19 April 2022). All the procedures and protocols followed in the current study were in accordance with the Declaration of Helsinki. Informed oral/written consent was obtained from the patient or guardian. A total of 70 subjects were recruited from different areas of Punjab, Pakistan, and divided into two groups: Healthy control (*n* = 39) and Patients (*n* = 31). Healthy controls were without any gastroenterological conditions or gluten sensitivity. Celiac disease was diagnosed by an expert gastroenterologist based on gluten sensitivity and gastrointestinal problems. A tissue transglutaminase IgA (tTG-IgA) antibody test as well as endoscopy (extension of the crypts, partial to complete villous atrophy according to Marsh classification [32]) were used to confirm the disease diagnosis. 

After informed consent, a 3 mL venous blood sample was aseptically drawn by an expert phlebotomist in an EDTA-containing vacutainer. The phenol-chloroform-isoamyl alcohol method was used to extract DNA for genetic analysis. In this process, 1000 mL of tris-HCL (20 mM) was taken in the labeled Eppendorf tubes, and then we added 400 mL of blood sample, which was thawed at room temperature. Centrifuge the eppendorf tube at 13,200 rpm for 10 min. The pellet was saved, and the supernatant was discarded. A total of 500 mL of tri-HCL was added to the eppendorf tube containing the pellet after centrifugation. The pellet needs to break in 500 mL of tris-HCL. After completely breaking the pellet, the eppendorf was centrifuged at 13,200 rpm for 5 min. This washing step needs to be repeated again and again until we obtain a whitish or pinkish pellet. The next step is incubation. In this step, 375 µL of 0.2 M sodium acetate, 50 µL of SDS (10%), and 20 µL of proteinase K were added to the eppendorf containing the washed white or pink pellet. The pellet needs to be broken again into all these components. Incubate this mixture at 56 °C for 2 h or at 37 °C overnight. After the incubation, the next step was the separation of DNA from cell debris and proteins by using PCI (25 phenols, 24 chloroforms, and 1 isoamyl alcohol). For this purpose, 130 mL of PCI was added to an eppendorf containing the incubated mixture. Eppendorf is centrifuged again at 13,200 rpm for 10 min. Two layers appeared after centrifugation: the upper aqueous layer contained the DNA threads, while the lower reddish organic layer contained the proteins and cell debris. The upper aqueous layer containing DNA was transferred to another labeled eppendorf tube very carefully without disturbing the lower reddish layer. The next step was washing the DNA with ethanol. For this purpose, 1 mL of ice-cold absolute was added to the eppendorf containing the aqueous layer, which was then centrifuged at 13,200 rpm for 10 min. After centrifugation, DNA stuck to the bottom as a tiny pellet, so very carefully remove all the ethanol without disturbing the pellet. To remove all the protein contaminants from DNA, 500 µL of 70% ethanol is added again to the eppendorf, followed by 10 min of 13,200 rpm centrifugation. Again, very carefully remove 500 µL of 70% ethanol from the eppendorf without disturbing the pellet. Carefully place the eppendrof on the tissue by rotating the tube at 180° so that any single droplet must be removed from the tube. After that, leave the open-lidded tube under the fan for proper air drying, so the remaining droplets must evaporate as well. Then, add 150 µL of sterile water to the tube and store the tube at −20 °C [33].

A questionnaire about demographic, clinical, and dietary details and medicinal history was also filled out for all enrolled subjects.

### 2.2. Genetic Analysis

The quality and quantity of extracted genomic DNA were assessed using 0.8% agarose gel electrophoresis and a NanoDrop^TM^ 2000c spectrophotometer (ND 2000, Thermo Fischer Scientific, Waltham, MA, USA). Agarose gel electrophoresis was run for 30 min at 90 volts.

Celiac disease (CD)-associated genetic variations of *LPP* and *ZMIZ1* were searched in various databases like NCBI Pubmed and Google Scholar by using various keywords like genetic variations, *LPP* gene polymorphism, genetics of celiac disease, *ZMIZ1* gene variations, and many more. A study of previously published literature summarized that *LPP* rs1464510 and *ZMIZ1* rs1250552 were associated with the risk of CD. The majority of the studies reported their positive association; however, a few studies also reported that these polymorphisms had no role in CD. Thus, these polymorphisms were selected to study their association with CD in the Pakistani population.

To analyze selected genetic variants (*LPP* rs1464510 and *ZMIZ1* rs1250552), primers were designed for tetra amplification refractory mutation system polymerase chain reaction (T-ARMS PCR) by following the steps mentioned by Hussain et al. [34]. Primer 1 software was used to design primers for our selected SNPs (*LPP* rs1464510 and *ZMIZ1* rs1250552). Primer 1 comes with different possible primer combinations for the specific SNP. All the possible combinations of Primers for a SNP were checked by an oligo-analyzer for different parameters like GC content, Tm, length of primer, secondary structures, hetro-dimers, and molecular weight. After keenly observing all these characteristics, the best primer pairs were selected, which were further used for genetic analysis. GC contents between 40 and 60% are recommended. A GC content of 40–60% is recommended. If there is a secondary structure between the set of tetra primers, significant dimer amplification occurs, but no amplification product is generated. To avoid this kind of complexity in primer design, the internal hairpin should be less than G of −3.3 kcal/mol, the external hairpin should be less than G of −2 kcal/mol, and self-dimers less than G of −6 kcal/mol are acceptable at the three prime ends of the primers. T-ARMS PCR is a convenient, less laborious, and cost-effective genotyping assay. It works by using two pairs of primers: (1) inner primers and (2) outer primers. Outer primers are not allele specific; however, inner primers are allele specific. T-ARMS PCR only needs gel electrophoresis after the PCR reaction. Thus, it reduces the time and cost involved in genotyping by Sanger sequencing without compromising the specificity and accuracy of genotypes. The sequence of primers used for genotyping *LPP* rs1464510 and *ZMIZ1* rs1250552 and the resulting product sizes are provided in Table 1.

**Figure 1:**
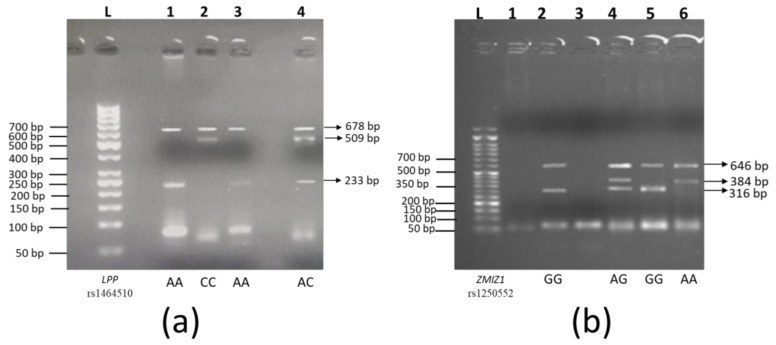
Gel electrophoresis of *LPP* rs1454510 and *ZMIZ1* rs1250552: (**a**) *LPP* rs1464510; Lane L shows a 100 bp ladder. Lanes 1 and 3 show bands at 678 bp and 233 bp, which indicate a homozygous AA genotype. Lane 2 indicates homozygous CC genotyping by showing bands at 678 bp and 509 bp. Lane 4 shows heterozygous AC genotype, as three bands are present at 678 bp, 509 bp, and 233 bp. (**b**) *ZMIZ1* rs1250552; lane L shows a 100 bp ladder. Lanes 1 and 3 did not show any amplification. Lanes 2 and 5 show homozygous GG genotype (646 bp and 316 bp bands). Lane 4 shows heterozygous AG genotypes (646 bp, 384 bp, and 316 bp bands). Lane 6 indicates a homozygous AA genotype (646 bp and 384 bp bands).

T-ARMS PCR primers were optimized for the genotyping of selected variations by using gradient PCR. After primer optimization, genotyping of all samples was carried out by T-ARMS-PCR using the primers enlisted in Table 1. Amplified products were resolved by horizontal gel electrophoresis using ethidium bromide added to a 1.5% agarose gel. A DNA ladder was used to estimate the size of the amplified product, and gel was visualized under UV light in the Gel Doc EZ system (Figure 1). 

### 2.3. Statistical Analysis

Statistical analysis was carried out by SPSS software, version 20 (IBM Inc., New York, NY, USA). Allelic frequency was calculated by the gene counting method, while inferential statistics such as chi-square were employed to calculate the genotype and determine the association of LPP rs1464510 and ZMIZ1 rs1250552 with the risk of celiac disease. Multinomial regression analysis was used to quantify the disease risk associated with the studied polymorphisms. Odds ratios and 95% confidence intervals were calculated by multinomial regression analysis, which was adjusted for age and gender. Genotype was taken as an independent variable. Various models were applied for regression analysis. The dominant model checked the combined effect of homozygous recessive and heterozygous genotypes on the risk of CD in comparison to the homozygous dominant genotype. The codominant model checked the individual effects of homozygous recessive and heterozygous genotypes on CD risk in comparison to the homozygous dominant genotype. The recessive model compared the combined effect of homozygous dominant and heterozygous genotypes with homozygous recessive genotypes. The allelic model compared both alleles of LPP rs1464510 and ZMIZ1 rs1250552. A α-error of 5% was assigned for statistical significance. A *p*-value of <0.05 was considered statistically significant. 

## 3. Results

LPP rs1464510 genotypic frequencies were significantly different between healthy controls and patients (χ^2^ = 6.033, *p* = 0.049) (Table 2). Intragroup analysis of genotypic frequencies showed that the LPP rs1464510 AA genotype is more prevalent in health controls (51%) as compared to heterozygous AC genotypes (39%) and homozygous CC genotypes (10%). Contrary to this, in patients, the LPP rs1464510 heterozygous AC genotype was more prevalent (61%) as compared to the homozygous AA genotype (23%). 

Intergroup analysis showed the opposite trend in frequencies of the AA and AC genotypes in the healthy control and patient groups. The frequency of the AA genotype was nearly double in healthy controls as compared to patients (51% vs. 23%), while the frequency of the AC genotype was higher in patients (61% vs. 39%). Allelic frequencies also showed significant differences in the prevalence of the A and C alleles between healthy controls and patients (χ^2^ = 4.421, *p* = 0.035). The frequency of the LPP rs1464510 A allele was higher in both study groups; however, it was more prevalent in the healthy group as compared to patients (71% vs. 53%) (Table 2). Higher frequencies of the LPP rs1464510 AA genotype and A allele in healthy controls could be an indication that this allele and genotype play a protective role for celiac disease. 

Genotypic frequencies of *ZMIZ1* rs1250552 were not significantly different between study groups (χ^2^ = 5.49, *p* = 0.064); however, allelic frequencies showed a border line difference (χ^2^ = 3.867, *p* = 0.049). In healthy controls, allelic frequencies of the *ZMIZ1* rs1250552 A and G alleles were the same, while in patients, the A allele was more prevalent (60%) than the G allele (40%). 

The association between celiac disease, *LPP* rs1464510, and *ZMIZ1* rs1250552 was further explored and quantified by odds ratio (Table 3). The odds ratio was calculated by applying multinomial regression adjusted for age and gender. Several models were applied to check the CD risk associated with genotypes of *LPP* rs1464510 and *ZMIZ1* rs1250552. Allelic model demonstrated that carriers of the *LPP* rs1464510 A allele have a 52% lower risk of developing CD as compared to carriers of the LPP rs1464510 C allele (OR 0.48, CI:0.24–0.96, *p* = 0.037). 

Furthermore, carriers of the *LPP* rs1464510 AA genotype also showed reduced risk of CD as compared to the carriers of the CC genotype in the co-dominant model (OR 0.28, CI: 0.058–1.35, *p* = 0.11) and dominant model (OR 0.68, CI: 0.15–2.60, *p* = 0.52); however, these results were not statistically significant. The LPP rs1464510 AC genotype showed a slightly higher risk of celiac disease in the co-dominant model (OR 1.01, CI: 0.23–4.45, *p* = 0.98). In line with these results, the recessive model demonstrated that rs1464510 C allele carriers are at 2.6 times higher risk of developing celiac disease as compared to the carriers of the AA genotype (OR 3.65, CI: 1.25–10.63, *p* = 0.017). 

For *ZMIZ1* rs1250552, the allelic model showed that carriers of the G allele have a reduced risk of CD in comparison to carriers of the A allele (OR 0.47, CI: 0.22–1.00, *p* = 0.051). However, these findings could not reach statistical significance. In comparison to the allelic model, the dominant model showed that AG+GG genotype carriers have a 73% reduced risk of CD (OR 0.27, CI: 0.09–0.83, *p* = 0.022). The *ZMIZ1* rs1250552 AG genotype also demonstrated a significant reduction in the onset of CD (OR 0.26, CI: 0.077–0.867, *p* = 0.028). While the recessive model exhibited an increased risk of CD in AA+AG genotype carriers, collectively, it could be said that the *LPP* rs1464510 A allele and the *ZMIZ1* rs1250552 G allele protect against CD and reduce the risk of CD development.

## 4. Discussion

The current study aimed to check the association of *LPP* rs1464510 C>A and ZMIZ1 rs1250552 A>G with the risk of celiac disease (CD) in the Pakistani population. To the best of our knowledge, this study is one of the first to explore the genetics of CD in Pakistani subjects. Previously published studies have mainly focused on the clinical side and challenges of gluten-free diets. 

The current study demonstrated that the *LPP* rs1464510 A allele is more prevalent in healthy subjects, and carriers of the A allele are at reduced risk of developing CD as compared to individuals carrying the C allele. While allelic frequency for *ZMIZ1* rs1250552 was the same in healthy controls for both alleles, the A allele was more prevalent in patients. Studied polymorphisms (*LPP* rs1464510 and *ZMIZ1* rs1250552) are intronic variants and could be influencing the expression of LPP and ZMIZ1 proteins, which play an important role in maintaining cell physiology and activating transcription, respectively. The *ZMIZ1* gene is also involved in immune function, which is central to the pathology of CD. LPP interacts with paxillin in focal adhesions and alters the cell shape and cytoskeleton arrangement in enterocytes. Microtubules also play an important role in maintaining the cell’s shape. A recent study by Stricker et al. also demonstrated that posttranslational modification of microtubules disturbs cell morphology and promotes CD [35]. Intact enterocytes are the primary requirement for proper digestion and absorption of nutrients. Nanayakkara et al. proved that gliadin (a type of gluten protein) peptides disturb the homeostasis of enterocytes by altering the cell shape, rearranging the cytoskeleton, increasing focal adhesions, and altering LPP cellular distribution [20]. LPP is found to be more strongly activated in the enterocytes of celiacs as compared to healthy cells [36]. This further strengthens the role of *LPP* in the onset of CD. 

Genetic studies have also shown that non-HLA genes also play a significant role in estimating the CD risk along with HLA genes (which account for 40% of the CD risk) [37]. Various immune-related genes are also associated with CD [38]. Sharma et al. have stated that the association of five non-HLA genes (*TAGAP*, *IL18R1*, *RGS21*, *PLEK*, and *CCR9*) with the risk of celiac disease varies with geographical differences [39]. Various studies conducted on first degree relatives and siblings have shown that genetic variations in non-HLA genes can help in the assessment of celiac disease. These non-HLA genetic variants can modulate the immune response to gluten. 

Most of the genome-wide association studies (GWAS) have been conducted on European populations; no study has addressed the association of genetic variants with the risk of celiac disease in Asian populations. Various GWAS have proved that rs1464510 gene polymorphism in non-HLA *LPP* genes is a strong predictor of celiac disease in Swedish families [27,40] and the US population [41]. However, in Italian families, *LPP* rs1464510 showed a moderate association with celiac disease [42]. Some studies have also shown the involvement of rs1464510 in other immune-related diseases like cancer, vitiligo, diabetes, and rheumatoid arthritis [43]. These co-morbidities are also influenced by the genetic variations of the *ZMIZ1* gene [44,45]. This gene is a strong risk predictor of vitiligo in the Chinese population. Celiac disease-associated changes in enterocytes also trigger variations in skin cells and lead to skin co-morbidities [46]. In line with the findings of previous studies, our study also concluded that *LPP* rs1464510 and ZMIZ1 rs1250552 are associated with CD. *LPP* rs1464510 A allele protects from celiac disease, while the C allele increases the risk of celiac disease.

This study provides insights into the genetics of celiac disease in the Asian population, and it is the first study from Pakistan reporting the association of the *LPP* rs1464510 A allele and the *ZMIZ1* rs1250552 AG genotype with the risk of celiac disease. This information can also help clinicians diagnose celiac disease, which is tricky owing to the overlapping symptoms with other diseases. Clinicians can also use this genetic information to assess the risk of celiac disease in non-symptomatic children of a family with a history of celiac disease. Although this study has added important information to the present literature, more studies with larger sample sizes and more stringent inclusion and exclusion criteria should be conducted to verify this association in the Punjabi Pakistani population. 

## 5. Conclusions

The current study summarizes that *LPP* rs1464510 and *ZMIZ1* rs1250552 are associated with the risk of celiac disease. The *LPP* rs1464510 A allele is more prevalent in the Punjabi Pakistani population. Statistical analysis revealed that the *LPP* rs1464510 A allele and the *ZMIZ1* rs1250552 AG genotype reduce the risk of celiac disease by 52% and 73%, respectively. *LPP* rs1464510 C allele increases the risk of celiac disease by 2.6 folds. 

## Figures and Tables

**Table 1:** Tetra ARMS-PCR primers for genotyping *LPP* rs1464510.

Primer Name	Primer Sequence (5′ to 3′)	Amplicon Size	Annealing Temperature
rs1464510 F inner	GTCCATAGATGTGATCCTGAAACTGATTTGAGAA	Control = 678 bp Allele A = 233 bp Allele C = 509 bp	66 °C
rs1464510 R inner	AATGGCAACACAGTAAAAATGAACCAGGGTG		
rs1464510 F outer	GGTGGTACTTATGGGAATACAGGCTTCAG		
rs1464510 R outer	CAAGCTACTCACTAGTTTTTGTAAGAGAGGCTAG		
rs1250552 F inner	GCAGGACAGAGATCTGCGAGAGAGACGG	Control = 646 bp Allele G = 316 bp Allele A = 384 bp	54 °C
rs1250552 R inner	GGAGGAGAGCCTCCTCCAGGGAACCT		
rs1250552 F outer	AAACAAGGAGAGGGAGAGGGGTTCCTGG		
rs1250552 R outer	ATATTTGGCTTGATGTCAGGCGGGAAGG		

**Table 2:** Genotypic and allelic frequency of LPP rs1464510 and ZMIZ1 rs1250552 in the population of Punjab, Pakistan.

Genetic Variants			Healthy Control N = 39	Patients N = 31	Significance
*LPP* rs1464510	Genotypes	AA	20 (51%)	7 (23%)	χ^2^ = 6.033 *p* = 0.049
		AC	15 (39%)	19 (61%)	
		CC	4 (10%)	5 (16%)	
	Alleles	A	55 (71%)	33 (53%)	χ^2^ = 4.421 *p* = 0.035
		C	23 (29%)	29 (47%)	
*ZMIZ1* rs1254552	Genotypes	AA	9(23%)	16(52%)	χ^2^ = 5.49 *p* = 0.064
		GG	9(23%)	10(32%)	
		AG	21(54%)	5(16%)	
	Alleles	A	39(50%)	37(60%)	χ^2^ = 3.867 *p* = 0.049
		G	39(50%)	25(40%)	

AA represents homozygous wild type, AC and AG are heterozygotes and CC and GG are homozygous mutant.

**Table 3:** Multinomial regression analysis of rs1464510 for the risk of celiac disease.

Genetic Variant	Model	Multinomial Regression Odds Ratio (95% Confidence Interval) *p*-Value	
*LPP* rs1464510	Co-dominant model	AA	0.28 (0.058–1.35) 0.11
		AC	1.01 (0.23–4.45) 0.98
		CC	Ref
	Recessive	CA+CC	3.65 (1.25–10.63) 0.017
		AA	Ref
	Dominant	CA+AA	0.68 (0.15–2.60) 0.52
		CC	Ref
	alleles	A	0.48 (0.24–0.96) 0.037
		C	Ref
*ZMIZ1* rs1250552	Co-dominant model	AA	Ref
		AG	0.26 (0.077–0.867) 0.028
		GG	0.31 (0.069–1.36) 0.121
	Recessive	GG	Ref
		AA+AG	1.56 (0.41–5.86) 0.52
	Dominant	AA	Ref
		AG+GG	0.27 (0.09–0.83) 0.022
	Alleles	A	Ref
		G	0.47 (0.22–1.00) 0.051

AA represents homozygous wild type, AC and AG are heterozygotes and CC and GG are homozygous mutant.

## Data Availability

The data presented in this study are available on request from the corresponding author.
